# Improving missing value imputation of microarray data by using spot quality weights

**DOI:** 10.1186/1471-2105-7-306

**Published:** 2006-06-16

**Authors:** Peter Johansson, Jari Häkkinen

**Affiliations:** 1 Computational Biology, Department of Theoretical Physics, Lund University, SE-223 62 Lund, Sweden

## Abstract

**Background:**

Microarray technology has become popular for gene expression profiling, and many analysis tools have been developed for data interpretation. Most of these tools require complete data, but measurement values are often missing A way to overcome the problem of incomplete data is to impute the missing data before analysis. Many imputation methods have been suggested, some naïve and other more sophisticated taking into account correlation in data. However, these methods are binary in the sense that each spot is considered either missing or present. Hence, they are depending on a cutoff separating poor spots from good spots. We suggest a different approach in which a continuous spot quality weight is built into the imputation methods, allowing for smooth imputations of all spots to larger or lesser degree.

**Results:**

We assessed several imputation methods on three data sets containing replicate measurements, and found that weighted methods performed better than non-weighted methods. Of the compared methods, best performance and robustness were achieved with the weighted nearest neighbours method (WeNNI), in which both spot quality and correlations between genes were included in the imputation.

**Conclusion:**

Including a measure of spot quality improves the accuracy of the missing value imputation. WeNNI, the proposed method is more accurate and less sensitive to parameters than the widely used kNNimpute and LSimpute algorithms.

## Background

During the last decade microarray technology has become an increasingly popular tool for gene expression profiling. Microarrays have been used in numerous biological contexts from studies of differentially expressed genes in tumours [1-4] to identification of cell cycle regulated genes in yeast [5]. A theme in microarray investigations is that they generate large amounts of data, and computer-based visualization and analysis tools must be used in experiment analysis. Tools such as hierarchical clustering [6], multidimensional scaling [7], and principal component analysis [8] are frequently used to visualize data. Machine learning methods like support vector machines [9] and artificial neural networks [10] have been used successfully to classify tumor samples. Common for these methods is that they in their standard versions assume complete data sets.

However, data is usually not complete. Data values may be missing due to poor printing of the arrays and consequently marked as missing during image analysis, but more common is that values are marked to be missing in a quality filtering pre-processing step. Common filter criteria are to mark spots with small area, spots with noisy background, spots with low intensity, or combinations of these [11]. One strategy to keep data complete is to remove reporters having missing values, but this may lead to an unnecessarily large loss of data. In particular when working with large data sets, reporters rarely have a complete set of values over all experiments. Another strategy is to keep reporters with not too many missing values and modify the subsequent analysis to handle incomplete data. However, it may not be feasible to modify the analysis tool, and therefore a popular approach is to impute the missing data in an intermediate step before analysis.

A common method to impute missing values is to replace missing values with the reporter average, *i.e*., the average for the particular reporter over all experiments. Troyanskaya *et al*. showed that this method is not sufficient as it neglects correlations in data [12]. They also suggested a method KNNimpute, that was shown to reconstruct missing values well. In KNNimpute, for each reporter the most similar reporters are found and the weighted average of these reporters is used as the imputation value. Other imputation methods have been suggested [13-18] using the same basic idea that the imputation value is taken as an average over the neighbouring reporters.

As far as we know, all suggested imputation methods are binary in the sense that each spot is considered either missing or present. Hence they depend on a cutoff, *e.g*., in intensity, separating poor spots from good spots. Tuning this cutoff is a balance act – a too liberal cutoff means noisy spots are kept in data, which may complicate subsequent analysis. On the other hand being too strict means spots containing information are marked as missing values and information is thrown away.

We suggest a more balanced approach, in which a spot quality weight is built into the imputation methods: good quality spots have more impact on the imputation of other spots, and are themselves subject to less imputation than spots with poorer quality. To examine the effects of this approach we extended two widely used methods, average imputation and KNNimpute [12], to handle continuous weights. We applied the two resulting methods to three data sets containing replicate measurements and found that weighted methods perform better that non-weighted.

## Results and discussion

As outlined in the Methods section, we devised two imputation methods using spot quality weights. These methods are generalizations of two non-weight based methods and we evaluated the methods with replicate data sets. We used the mean squared deviation (MSD) to compare the performance of the two suggested methods, their non-weighted counterparts, and LSimpute [17]. We did the comparisons varying the spot quality threshold for missing values for the non-weight based methods. Correspondingly, for the weighted methods we varied the weight tuning parameter *β *in the calculations of the weights (see Methods).

**Figure 1:**
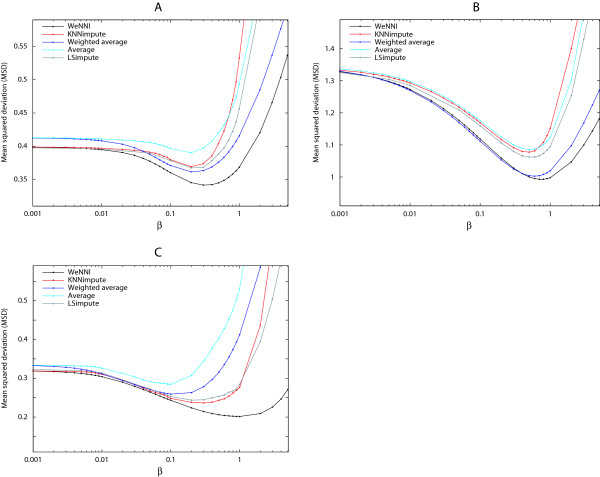
**WeNNI is the most accurate imputation method**. Performance of the five imputation methods with varying *β*. As explained in the Methods section, larger *β *changes weights to smaller values. In non-weighted methods *β *is the SNR cutoff. The increase in MSD for large *β *is an effect from too many missing values, which implies imputation breaks down. The standard error of means are within the line thicknesses. (A) *Breast cancer data*. WeNNI (black line) has the lowest MSD and the weighted methods perform better than the non-weighted methods. All methods have a minimum MSD around *β *= 0.2. (B) *Melanoma data*. WeNNI (black line) has the lowest MSD and the weighted methods perform better than the non-weighted methods. All methods have a minimum MSD around *β *= 0.6. (C) *Mycorrhiza data*. WeNNI (black line) retains the lowest MSD, whereas KNNimpute (red line) performs better that the weighted reporter average method. This may be explained as an effect of a different experimental design as discussed in the text. The minimum MSD is found in a *β *range 0.3–1 for the different methods.

In Figure 1 we present how the performance varied with a changing *β *in the three data sets. The plots show that WeNNI has the lowest MSD for all three data sets, the weighted methods outperform their non-weighted counterparts, and the minimum MSD is within the *β *range 0.1–1 for all methods.

The overall MSD is larger for the melanoma data set compared to the two other data sets, which may be due to that the melanoma data was generated a few years earlier than the other data.

An interesting finding was that weighted reporter average outperformed KNNimpute and LSimpute in the breast cancer and melanoma data sets. This result was unexpected since the weighted reporter average method neglects correlations between reporters. Moreover, the assumption for using reporter average is in general problematic, since the expression of a reporter in one experimental condition does not always reflect the expression of the reporter in another condition. For the mycorrhiza data used here the situation is even worse, since the cyclic experimental design [20] makes the expression value in one experiment auti-correlated to the reporter's average over the other experiments. For the nearest neighbours imputation methods however this problem does not arise because imputations are calculated as an average over the same experiment. These results imply one should consider the experimental design and choose imputation method carefully.

For small *β *all methods showed approximately equal performance. This result was expected, because for small *β *most weights are close to unity. In consequence, only a small fraction of the spots are imputed and make a minor contribution to the MSD. Moreover, the weights are effectively binary for small *β*, and the weighted methods become identical to their non-weighted counterparts.

**Figure 2:**
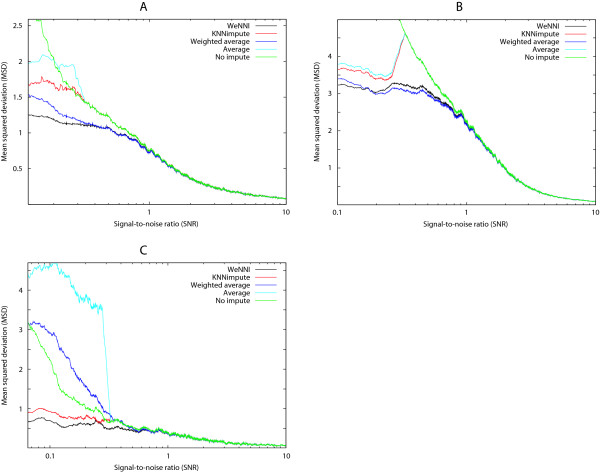
**WeNNI is most accurate over all ranges of spot quality**. The contribution to MSD for specific SNR for the different imputation methods applied to the three different data sets using *β *= 0.3. These plots were created using a sliding window containing 1% of all spots. Spots with small SNR (low quality) have the largest impact on MSD. (A) In the *breast cancer data *a weighted scheme is clearly essential and WeNNI is most accurate over all ranges of SNR (B) In the *melanoma data *a weighted scheme is clearly essential and the weighted reporter average show best performance for an SNR range 0.2–1. (C) In the *mycorrhiza data *the breakdown of the average reporter methods is very prominent. For the SNR range 0.07–0.4 it is even better to use no impute (green line) than the average methods. The breakdown of the reporter average methods are discussed in the text.

To examine the difference between the weighted methods and their non-weighted counterparts, we plotted MSD as a function of SNR (Figure 2). As expected, spots with small SNR contributed most to MSD. The discrepancy between mycorrhiza data and the other two data sets also showed up here – the breakdown of the reporter average methods in the mycorrhiza data is very prominent (Figure 2C). The melanoma and breast cancer data showed very similar patterns for the different methods and the weighted methods performed better than their counterparts for all SNR. In some ranges of SNR, weighted reporter average even surpassed WeNNI, but overall WeNNI imputed the values most accurately.

**Figure 3:**
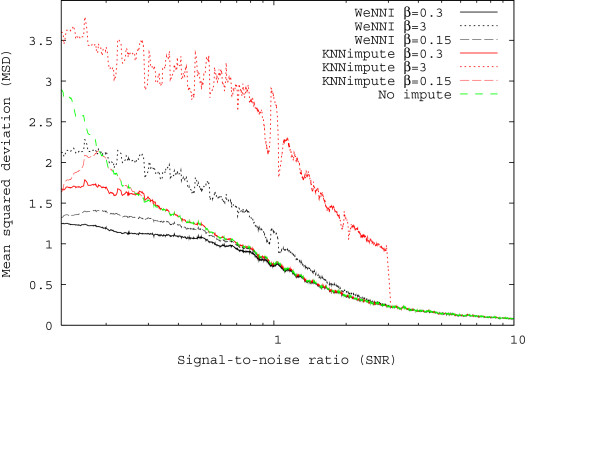
**Comparison of WeNNI and KNNimpute**. MSD contributions from specific SNR and different *β *for the *breast cancer data *set. This plot was created using a sliding window containing 1% of all spots.

In Figure 3, we demonstrate the effect of varying *β *for WeNNI and KNNimpute using the breast cancer data. In KNNimpute, only spots with smaller SNR than the cutoff *β *are imputed, and consequently the performance for SNR larger than *β *follows the no impute curve. For KNNimpute a choice of *β *= 0.3 was close to optimal. Using a smaller *β *deteriorated the imputation in two ways. Spots with SNR between the used *β *and the optimal value 0.3 were not imputed. In the plot we can see that the quality of these spots is so bad that preferably they should be imputed. More importantly, since these spots were not considered missing they were used in the imputation of values with very small SNR, which made the imputation less accurate. Moreover, when we used a too large *β*, the spots with SNR in the range 0.3–3 were imputed and their deviation from the replicate became larger than if they were not imputed. Also, the imputation of the spots with very small SNR became worse, since less information was used in the imputation. Choosing *β *corresponds to setting a cutoff in quality control criteria, and Figure 3 illustrates how a suboptimal cutoff level will lead to less reliable data. For WeNNI the cutoff is smoothened by the usage of continuous weights, and consequently WeNNI is more robust with respect to *β*.

**Figure 4:**
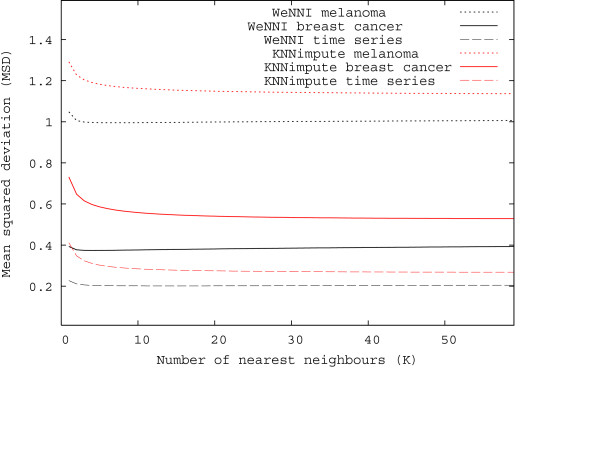
**WeNNI and KNNimpute are insensitive to number of neighbours used**. Performance of WeNNI and KNNimpute is plotted against the number of nearest neighbours for all three data sets using *β *= 1.

In Figure 4, we illustrate how the performance of WeNNI and KNNimpute depends on the number of neighbours, *K*, used in the imputation. We notice that both methods are insensitive to changing *K*. For a small number of neighbours, both methods are insufficient. Troyanskaya *et al*. suggested *K *to be in the range between 10 and 20 neighbours for KNNimpute [12]. Our results agree with this finding and also show that the imputation of our data sets was accurate for a larger number of nearest neighbours.

When comparing non-weighted imputation methods, it is natural to calculate the comparison measure over imputed values only. Including non-imputed values in the evaluation makes no sense, as these values are not modified and thus independent of the imputation method. This is also the way imputation methods are compared in the literature [12-18]. However, for a weighted method every expression value is modified, and it is sensible to include all values in the calculation of MSD. In Table 1 we compare LSimpute.

KNNimpute, and WeNNI using both MSD and MSD_imputed. MSD_imputed is calculated as MSD but over imputed values (as defined by binary methods) only. We note that MSD_imputed is larger than MSD for all methods and data sets, which is expected because MSD_imputed is calculated over poor spots only and poor spots are expected to deviate more from their duplicates. Moreover, for MSD_imputed the difference between the methods is more apparent, which is a consequence from comparing poor spots only. In MSD all spots are included in the comparison and as good quality spots are modified to lesser degree, the difference between the methods looks smaller. We note that WeNNI is the most accurate method also using MSD_impute, in other words. WeNNI has the best performance even when values not imputed by non-weighted methods are excluded from the comparison.

For the largest data set, *breast cancer data *with approximately 55000 spots, a typical WeNNI run takes approximately 10 CPU minutes on a off-the-shelf computer (AMD Athlon 3700+ processor and 1 GB RAM), whereas kNNI is twice as fast. These two algorithms are implemented in the same C++ code base and differs only in the calculation of the imputation values. A comparison with LSimpute is not fair since LSimpute is an adaptive algorithm and is implemented in Java.

### Spot quality weights and expression value imputation

The starting point for imputing expression values in this report is that the weight of a spot should depend on its quality, as best estimated from data. Here, we used a straight forward SNR based weight as it was not our aim to study quality of spots. The SNR based quality weights were introduced in [21], and many different studies of quality measures have been described [11,26-28]. These papers concentrate on studying how the quality of spots should be defined.

Analyses in microarray projects are commonly based on spot intensities, and for that reason we examined if using intensities instead of SNR changes the findings in this paper. We found that using this simpler quality weight (Eq. 4), the performance was almost as good as when using the SNR based weights (data not shown). The fact that the imputed expression value on average gets closer to its pristine replicate value, indicates that the SNR based weight may be a slightly better estimate of the spot quality.

In imputation of expression values, as in any transformation of data (*e.g*., LOWESS normalisation or centralisation), one must be careful to not destroy the biological signal in the data. In our three data sets, we noticed that when WeNNI is used, the deviation from the pristine replicate is on average smaller than when not doing the transformation, in other world, on average an expression value is closer to its replicate after the transformation. The effect is measurable even for the naïve weight used here.

The goal of a weight is to catch the "true" quality of the spot, and as such it is important to define spot quality weight calculation to suit the data at hand, prior knowledge, and expertise. One important aspect of applying prior knowledge into weight calculation is that initial pre-screening of array data should still be done before imputation, or any subsequent analysis. In this screening step bad spots are removed, and known malfunction in data (arrays) should be communicated with zero weights.

## Conclusion

Virtually every analysis of microarray data is preceded by a filtering step, in which each spot is required to fulfil certain quality control criteria. If the spot fails to meet the quality requirements it is marked as a missing value. This is equivalent to accompanying each expression value with a binary weight, and enforces an abrupt cutoff in quality control criteria. We have generalised two widely used imputation methods to use continuous weights. Our finding that the weighted imputation methods outperformed their non-weighted counterparts, suggests that using continuous weights is superior to using binary weights. Our suggested improvement – to use continuous weights – is generic in the sense that most imputation methods can be generalised to use continuous weights.

The weighted nearest neighbours imputation method presented in this paper. WeNNI, outperformed all other tested methods for the three different data sets used in this study. WeNNI performs accurate imputation of expression values and is insensitive to the parameter values used, *i.e*., the number of nearest neighbours and *β*. An increasing *β *corresponds to having a more strict spot quality control criteria. For a non-weighted method it means that more values are considered missing and consequently imputed. Our results suggest that the usage of a continuous weight makes the imputation less sensitive to the choice of *β*. The findings in this manuscript are based on comparisons of replicate data, however replicate data may not be available in every experimental setting and the scientific investigator cannot evaluate the impact of different parameter values. The results in this study show that the choice of parameters is not crucial, and suggest a value around 10 for nearest, neighbours and a *β *in the range 0.1–1.

## Methods

### Data sets and pre-processing

To evaluate the imputation methods, we used three data sets, i) *Melanoma data*. The melanoma data set was obtained from a panel of 61 human cell lines [19]. For each experiment, 19,200 reporters were printed in duplicates. Identification of individual spots on scanned arrays was done with ImaGene 4.0 (BioDiscovery, E1 Segundo, CA, USA). ii) *Breast cancer data*. The breast cancer data set is a subset of a larger ongoing study. We selected the 55 experiments that had been hybridised at the Swegene DNA Microarray Resource Centre in Lund, Sweden, and were from tumours mutated either in *BRCA1 *or in *BRCA2*. Each array contained 55,488 spots and except a small number of control spots each reporter was printed in duplicate. Identification of individual spots on scanned arrays was done with GenePix Pro 4.0 (Axon Instruments, Union City, CA, USA). iii) *Mycorrhiza data*. The mycorrhiza data set was generated to study ectomycorrhizal root tissue [20]. In order to avoid any bias from using dye swap replicates, we used half of the arrays from the study. We used the 10 arrays denoted R3 between ECM's at different time points, and R1 between ECM and REF (Figure 2 in [20]). Each array contained 10,368 spots and except a small number of control spots each reporter was printed four times. Identification of individual spots on scanned arrays was done with GenePix Pro 3.0.6.89) (Axon Instruments, Union City, CA, USA).

For each spot, we used the mean spot intensity, *I*_fg_, the mean background intensity, *I*_bg_, and the standard deviation of the background intensity, *σ*_bg_. For each spot we calculated the signal-to-noise ratio (SNR) [11] as

1SNR2=1SNRt2+1SNRc2=σbg,t2(Ifg,t−Ibg,t)2+σbg,c2(Ifg,c−Ibg,c)2.     (1)
 MathType@MTEF@5@5@+=feaafiart1ev1aaatCvAUfKttLearuWrP9MDH5MBPbIqV92AaeXatLxBI9gBaebbnrfifHhDYfgasaacH8akY=wiFfYdH8Gipec8Eeeu0xXdbba9frFj0=OqFfea0dXdd9vqai=hGuQ8kuc9pgc9s8qqaq=dirpe0xb9q8qiLsFr0=vr0=vr0dc8meaabaqaciaacaGaaeqabaqabeGadaaakeaafaqadeGabaaabaWaaSaaaeaacqaIXaqmaeaaieaacqWFtbWucqWFobGtcqWFsbGudaahaaWcbeqaaiabikdaYaaaaaGccqGH9aqpdaWcaaqaaiabigdaXaqaaiab=nfatjab=5eaojab=jfasnaaDaaaleaacqWF0baDaeaacqaIYaGmaaaaaOGaey4kaSYaaSaaaeaacqaIXaqmaeaacqWFtbWucqWFobGtcqWFsbGudaqhaaWcbaGae83yamgabaGaeGOmaidaaaaaaOqaaiabg2da9maalaaabaacciGae43Wdm3aa0baaSqaaiab=jgaIjab=DgaNjabcYcaSiab=rha0bqaaiabikdaYaaaaOqaaiabcIcaOiabdMeajnaaBaaaleaacqWFMbGzcqWFNbWzcqGGSaalcqWF0baDaeqaaOGaeyOeI0IaemysaK0aaSbaaSqaaiab=jgaIjab=DgaNjabcYcaSiab=rha0bqabaGccqGGPaqkdaahaaWcbeqaaiabikdaYaaaaaGccqGHRaWkdaWcaaqaaiab+n8aZnaaDaaaleaacqWFIbGycqWFNbWzcqGGSaalcqWFJbWyaeaacqaIYaGmaaaakeaacqGGOaakcqWGjbqsdaWgaaWcbaGae8NzayMae83zaCMaeiilaWIae83yamgabeaakiabgkHiTiabdMeajnaaBaaaleaacqWFIbGycqWFNbWzcqGGSaalcqWFJbWyaeqaaOGaeiykaKYaaWbaaSqabeaacqaIYaGmaaaaaOGaeiOla4caaiaaxMaacaWLjaWaaeWaaeaacqaIXaqmaiaawIcacaGLPaaaaaa@78B3@

Subscripts t and c denotes treatment and control, respectively. As expression value, *x*, we used the logarithm to base 2 of the ratio of the signal in the treatment sample and the signal in the control sample

x=log⁡2(Ifg,t−Ibg,cIfg,c−Ibg,c),     (2)
 MathType@MTEF@5@5@+=feaafiart1ev1aaatCvAUfKttLearuWrP9MDH5MBPbIqV92AaeXatLxBI9gBaebbnrfifHhDYfgasaacH8akY=wiFfYdH8Gipec8Eeeu0xXdbba9frFj0=OqFfea0dXdd9vqai=hGuQ8kuc9pgc9s8qqaq=dirpe0xb9q8qiLsFr0=vr0=vr0dc8meaabaqaciaacaGaaeqabaqabeGadaaakeaacqWG4baEcqGH9aqpcyGGSbaBcqGGVbWBcqGGNbWzdaWgaaWcbaGaeGOmaidabeaakmaabmaabaWaaSaaaeaacqWGjbqsdaWgaaWcbaacbaGae8NzayMae83zaCMaeiilaWIae8hDaqhabeaakiabgkHiTiabdMeajnaaBaaaleaacqWFIbGycqWFNbWzcqGGSaalcqWFJbWyaeqaaaGcbaGaemysaK0aaSbaaSqaaiab=zgaMjab=DgaNjabcYcaSiab=ngaJbqabaGccqGHsislcqWGjbqsdaWgaaWcbaGae8NyaiMae83zaCMaeiilaWIae83yamgabeaaaaaakiaawIcacaGLPaaacqGGSaalcaWLjaGaaCzcamaabmaabaGaeGOmaidacaGLOaGaayzkaaaaaa@551E@

where spots with non-positive signal in either treatment or control were marked as invalid.

We applied a liberal filter to the data. In the melanoma data set we kept reporters having less than 50% invalid values in both duplicates. The remaining data was split into two replicate data sets. This was also done for the two other data sets, with the exception that the mycorrhiza data was split into four replicate data sets. Each data set was then centralised experiment by experiment such that the average expression value for an experiment was zero.

After filtering, the melanoma data consisted of two replicate data sets each having 61 experiments and 17, 549 reporters, the breast cancer data consisted of two explicate data sets each having 55 experiments and 23,764 reporters, and the mycorrhiza data consisted of four replicate data sets each having 10 experiments and 2,052 reporters.

### Quality weight

The basis for weight calculations are two weight formulae inspired by previous work [21-24]. We used an SNR based weight defined as

w=11+β2SNRt2+β2SNRc2.     (3)
 MathType@MTEF@5@5@+=feaafiart1ev1aaatCvAUfKttLearuWrP9MDH5MBPbIqV92AaeXatLxBI9gBaebbnrfifHhDYfgasaacH8akY=wiFfYdH8Gipec8Eeeu0xXdbba9frFj0=OqFfea0dXdd9vqai=hGuQ8kuc9pgc9s8qqaq=dirpe0xb9q8qiLsFr0=vr0=vr0dc8meaabaqaciaacaGaaeqabaqabeGadaaakeaacqWG3bWDcqGH9aqpdaWcaaqaaiabigdaXaqaaiabigdaXiabgUcaRmaalaaabaacciGae8NSdi2aaWbaaSqabeaacqaIYaGmaaaakeaaieaacqGFtbWucqGFobGtcqGFsbGudaqhaaWcbaGae4hDaqhabaGaeGOmaidaaaaakiabgUcaRmaalaaabaGae8NSdi2aaWbaaSqabeaacqaIYaGmaaaakeaacqGFtbWucqGFobGtcqGFsbGudaqhaaWcbaGae43yamgabaGaeGOmaidaaaaaaaGccqGGUaGlcaWLjaGaaCzcamaabmaabaGaeG4mamdacaGLOaGaayzkaaaaaa@492D@

This weight is defined to be bound within zero and unity. The free parameter *β *is used to tune the distribution of weights. For a small *β *all weights are close to unity, except when zero or negative intensities have been measured which implies a zero weight. For a large *β *all weights are close to zero. In non-weighted (binary) methods we marked expression values to be missing when the corresponding continuous weight was less than 0.5. In this way *β *defined a cutoff for when a value is considered to be missing.

To cross check that the findings in this paper do not depend on SNR, we also used a simple weight based on intensity only:

w=11+β2(Ifg,t−Ibg,t)2+β2(Ifg,c−Ibg,c)2.     (4)
 MathType@MTEF@5@5@+=feaafiart1ev1aaatCvAUfKttLearuWrP9MDH5MBPbIqV92AaeXatLxBI9gBaebbnrfifHhDYfgasaacH8akY=wiFfYdH8Gipec8Eeeu0xXdbba9frFj0=OqFfea0dXdd9vqai=hGuQ8kuc9pgc9s8qqaq=dirpe0xb9q8qiLsFr0=vr0=vr0dc8meaabaqaciaacaGaaeqabaqabeGadaaakeaacqWG3bWDcqGH9aqpdaWcaaqaaiabigdaXaqaaiabigdaXiabgUcaRmaalaaabaacciGae8NSdi2aaWbaaSqabeaacqaIYaGmaaaakeaacqGGOaakcqWGjbqsdaWgaaWcbaacbaGae4NzayMae43zaCMaeiilaWIae4hDaqhabeaakiabgkHiTiabdMeajnaaBaaaleaacqGFIbGycqGFNbWzcqGGSaalcqGF0baDaeqaaOGaeiykaKYaaWbaaSqabeaacqaIYaGmaaaaaOGaey4kaSYaaSaaaeaacqWFYoGydaahaaWcbeqaaiabikdaYaaaaOqaaiabcIcaOiabdMeajnaaBaaaleaacqGFMbGzcqGFNbWzcqGGSaalcqGFJbWyaeqaaOGaeyOeI0IaemysaK0aaSbaaSqaaiab+jgaIjab+DgaNjabcYcaSiab+ngaJbqabaGccqGGPaqkdaahaaWcbeqaaiabikdaYaaaaaaaaOGaeiOla4IaaCzcaiaaxMaadaqadaqaaiabisda0aGaayjkaiaawMcaaaaa@5D7B@

This weight is also bound to be within zero and unity, and *β *has the same function here as for the SNR based weight above.

### Imputation methods

We compared five imputation methods; three non-weight based methods, reporter average, KNNimpute, and LSimpute_adaptive; and two weight based, weighted reporter average and weighted nearest neighbours imputation (WeNNI).

#### Reporter average methods

The widely used reporter average imputation method is intuitive and easy to implement. Assuming the expression level of a reporter in one experiment to be similar to the expression level in other experiments the expression value is imputed as the average of the reporter's expression value over all experiments. Similarly to Andersson *et al*. [21], we extended the reporter average by using continuous spot quality weights between zero and unity. A spot with a weight equal to unity is not imputed, whereas for a spot with weight equal to zero the expression value is imputed to be the weighted reporter average. A spot having an intermediate weight is imputed as a linear combination of the extreme cases above. These three cases are covered in the imputation equation

x′re
 MathType@MTEF@5@5@+=feaafiart1ev1aaatCvAUfKttLearuWrP9MDH5MBPbIqV92AaeXatLxBI9gBaebbnrfifHhDYfgasaacH8akY=wiFfYdH8Gipec8Eeeu0xXdbba9frFj0=OqFfea0dXdd9vqai=hGuQ8kuc9pgc9s8qqaq=dirpe0xb9q8qiLsFr0=vr0=vr0dc8meaabaqaciaacaGaaeqabaqabeGadaaakeaacuWG4baEgaqbamaaBaaaleaacqWGYbGCcqWGLbqzaeqaaaaa@311D@ = *w*_*re*_*x*_*re *_+ (1 - *w*_*re*_)x^re
 MathType@MTEF@5@5@+=feaafiart1ev1aaatCvAUfKttLearuWrP9MDH5MBPbIqV92AaeXatLxBI9gBaebbnrfifHhDYfgasaacH8akY=wiFfYdH8Gipec8Eeeu0xXdbba9frFj0=OqFfea0dXdd9vqai=hGuQ8kuc9pgc9s8qqaq=dirpe0xb9q8qiLsFr0=vr0=vr0dc8meaabaqaciaacaGaaeqabaqabeGadaaakeaacuWG4baEgaqcamaaBaaaleaacqWGYbGCcqWGLbqzaeqaaaaa@3121@,     (5)

in which *x*_*re *_is the expression value in reporter *r *and experiment *e*, *w*_*re *_is the quality weight, and x^re
 MathType@MTEF@5@5@+=feaafiart1ev1aaatCvAUfKttLearuWrP9MDH5MBPbIqV92AaeXatLxBI9gBaebbnrfifHhDYfgasaacH8akY=wiFfYdH8Gipec8Eeeu0xXdbba9frFj0=OqFfea0dXdd9vqai=hGuQ8kuc9pgc9s8qqaq=dirpe0xb9q8qiLsFr0=vr0=vr0dc8meaabaqaciaacaGaaeqabaqabeGadaaakeaacuWG4baEgaqcamaaBaaaleaacqWGYbGCcqWGLbqzaeqaaaaa@3121@ is the weighted reporter average

x^re=∑i=1Mwrixri∑i=1Mwri,     (6)
 MathType@MTEF@5@5@+=feaafiart1ev1aaatCvAUfKttLearuWrP9MDH5MBPbIqV92AaeXatLxBI9gBaebbnrfifHhDYfgasaacH8akY=wiFfYdH8Gipec8Eeeu0xXdbba9frFj0=OqFfea0dXdd9vqai=hGuQ8kuc9pgc9s8qqaq=dirpe0xb9q8qiLsFr0=vr0=vr0dc8meaabaqaciaacaGaaeqabaqabeGadaaakeaacuWG4baEgaqcamaaBaaaleaacqWGYbGCcqWGLbqzaeqaaOGaeyypa0ZaaSaaaeaadaaeWaqaaiabdEha3naaBaaaleaacqWGYbGCcqWGPbqAaeqaaOGaemiEaG3aaSbaaSqaaiabdkhaYjabdMgaPbqabaaabaGaemyAaKMaeyypa0JaeGymaedabaGaemyta0eaniabggHiLdaakeaadaaeWaqaaiabdEha3naaBaaaleaacqWGYbGCcqWGPbqAaeqaaaqaaiabdMgaPjabg2da9iabigdaXaqaaiabd2eanbqdcqGHris5aaaakiabcYcaSiaaxMaacaWLjaWaaeWaaeaacqaI2aGnaiaawIcacaGLPaaaaaa@511F@

where *M *is the number of experiments.

The use of the spot quality weight is twofold. First, the weight is used in the calculation of the reporter average. Second, the weight is used in the calculation of the imputed expression value – poor quality spots are changed more than good quality spots.

#### KNNimpute

The currently most popular imputation method that goes beyond reporter averaging. KNNimpute, has been shown to be a very good method for imputation of missing values [12]. The main idea of KNNimpute is to look for the *K *most similar reporters when a value is missing for a reporter. Two reporters *n *and *m *are considered to be similar when the Euclidean distance.

dnm2=1M∑i=1M(xni−xmi)2,     (7)
 MathType@MTEF@5@5@+=feaafiart1ev1aaatCvAUfKttLearuWrP9MDH5MBPbIqV92AaeXatLxBI9gBaebbnrfifHhDYfgasaacH8akY=wiFfYdH8Gipec8Eeeu0xXdbba9frFj0=OqFfea0dXdd9vqai=hGuQ8kuc9pgc9s8qqaq=dirpe0xb9q8qiLsFr0=vr0=vr0dc8meaabaqaciaacaGaaeqabaqabeGadaaakeaacqWGKbazdaqhaaWcbaGaemOBa4MaemyBa0gabaGaeGOmaidaaOGaeyypa0ZaaSaaaeaacqaIXaqmaeaacqWGnbqtaaWaaabCaeaacqGGOaakcqWG4baEdaWgaaWcbaGaemOBa4MaemyAaKgabeaakiabgkHiTiabdIha4naaBaaaleaacqWGTbqBcqWGPbqAaeqaaOGaeiykaKYaaWbaaSqabeaacqaIYaGmaaaabaGaemyAaKMaeyypa0JaeGymaedabaGaemyta0eaniabggHiLdGccqGGSaalcaWLjaGaaCzcamaabmaabaGaeG4naCdacaGLOaGaayzkaaaaaa@4D0F@

between their expression patterns is small. These *K *reporters are used to calculate a weighted average of the values in the experiment of interest. The weighted average is calculated as

x^re=∑i=1Kxiedri∑i=1K1dri,     (8)
 MathType@MTEF@5@5@+=feaafiart1ev1aaatCvAUfKttLearuWrP9MDH5MBPbIqV92AaeXatLxBI9gBaebbnrfifHhDYfgasaacH8akY=wiFfYdH8Gipec8Eeeu0xXdbba9frFj0=OqFfea0dXdd9vqai=hGuQ8kuc9pgc9s8qqaq=dirpe0xb9q8qiLsFr0=vr0=vr0dc8meaabaqaciaacaGaaeqabaqabeGadaaakeaacuWG4baEgaqcamaaBaaaleaacqWGYbGCcqWGLbqzaeqaaOGaeyypa0ZaaSaaaeaadaaeWaqaamaalaaabaGaemiEaG3aaSbaaSqaaiabdMgaPjabdwgaLbqabaaakeaacqWGKbazdaWgaaWcbaGaemOCaiNaemyAaKgabeaaaaaabaGaemyAaKMaeyypa0JaeGymaedabaGaem4saSeaniabggHiLdaakeaadaaeWaqaamaalaaabaGaeGymaedabaGaemizaq2aaSbaaSqaaiabdkhaYjabdMgaPbqabaaaaaqaaiabdMgaPjabg2da9iabigdaXaqaaiabdUealbqdcqGHris5aaaakiabcYcaSiaaxMaacaWLjaWaaeWaaeaacqaI4aaoaiaawIcacaGLPaaaaaa@51C5@

where *x*_*ie *_is the value of the *i*th nearest reporter, *d*_*ri *_is the distance between reporter *r *and reporter *i *and *K *is the number of neighbours to use in the calculation. This weighted average is used as imputation value of missing values.

#### Weighted Nearest Neighbours Imputation [WeNNI]

KNNimpute is binary in the sense that each value is regarded as either missing or present. In WeNNI, we smooth out this sharp border between missing and present values by assigning a continuous quality weight to each value, where a zero weight means the value is completely missing and a larger weight means the value is more reliable. In the special case when all weights are either 0 or 1. WeNNI is equivalent to KNNimpute.

The WeNNI method consists of two steps. First, we calculate distances between the reporters taking the weights into account. Second, we calculate a weighted average of the values of the nearest neighbours. We expanded the Euclidean distance used in KNNimpute to include quality weights. The weights were included in such a way that spots with large weights are more important for the distance measure than spots with low weights. We calculated the distance *d*_*nm *_between reporter *n *and reporter *m *as

dnm2=∑i=1Mwniwmi(xni−xmi)2∑i=1Mwniwmi,     (9)
 MathType@MTEF@5@5@+=feaafiart1ev1aaatCvAUfKttLearuWrP9MDH5MBPbIqV92AaeXatLxBI9gBaebbnrfifHhDYfgasaacH8akY=wiFfYdH8Gipec8Eeeu0xXdbba9frFj0=OqFfea0dXdd9vqai=hGuQ8kuc9pgc9s8qqaq=dirpe0xb9q8qiLsFr0=vr0=vr0dc8meaabaqaciaacaGaaeqabaqabeGadaaakeaacqWGKbazdaqhaaWcbaGaemOBa4MaemyBa0gabaGaeGOmaidaaOGaeyypa0ZaaSaaaeaadaaeWaqaaiabdEha3naaBaaaleaacqWGUbGBcqWGPbqAaeqaaOGaem4DaC3aaSbaaSqaaiabd2gaTjabdMgaPbqabaGccqGGOaakcqWG4baEdaWgaaWcbaGaemOBa4MaemyAaKgabeaakiabgkHiTiabdIha4naaBaaaleaacqWGTbqBcqWGPbqAaeqaaOGaeiykaKYaaWbaaSqabeaacqaIYaGmaaaabaGaemyAaKMaeyypa0JaeGymaedabaGaemyta0eaniabggHiLdaakeaadaaeWaqaaiabdEha3naaBaaaleaacqWGUbGBcqWGPbqAaeqaaOGaem4DaC3aaSbaaSqaaiabd2gaTjabdMgaPbqabaaabaGaemyAaKMaeyypa0JaeGymaedabaGaemyta0eaniabggHiLdaaaOGaeiilaWIaaCzcaiaaxMaadaqadaqaaiabiMda5aGaayjkaiaawMcaaaaa@62DB@

where *M *is the number of experiments. A weighted average of the nearest neighbours is calculated as

x^re=∑i=1Lwiexiedri∑i=1Lwiedri,     (10)
 MathType@MTEF@5@5@+=feaafiart1ev1aaatCvAUfKttLearuWrP9MDH5MBPbIqV92AaeXatLxBI9gBaebbnrfifHhDYfgasaacH8akY=wiFfYdH8Gipec8Eeeu0xXdbba9frFj0=OqFfea0dXdd9vqai=hGuQ8kuc9pgc9s8qqaq=dirpe0xb9q8qiLsFr0=vr0=vr0dc8meaabaqaciaacaGaaeqabaqabeGadaaakeaacuWG4baEgaqcamaaBaaaleaacqWGYbGCcqWGLbqzaeqaaOGaeyypa0ZaaSaaaeaadaaeWaqaamaalaaabaGaem4DaC3aaSbaaSqaaiabdMgaPjabdwgaLbqabaGccqWG4baEdaWgaaWcbaGaemyAaKMaemyzaugabeaaaOqaaiabdsgaKnaaBaaaleaacqWGYbGCcqWGPbqAaeqaaaaaaeaacqWGPbqAcqGH9aqpcqaIXaqmaeaacqWGmbata0GaeyyeIuoaaOqaamaaqadabaWaaSaaaeaacqWG3bWDdaWgaaWcbaGaemyAaKMaemyzaugabeaaaOqaaiabdsgaKnaaBaaaleaacqWGYbGCcqWGPbqAaeqaaaaaaeaacqWGPbqAcqGH9aqpcqaIXaqmaeaacqWGmbata0GaeyyeIuoaaaGccqGGSaalcaWLjaGaaCzcamaabmaabaGaeGymaeJaeGimaadacaGLOaGaayzkaaaaaa@5A6F@

where *L *is defined by

∑i=1Lwie≤K<∑i=1L+1wie.     (11)
 MathType@MTEF@5@5@+=feaafiart1ev1aaatCvAUfKttLearuWrP9MDH5MBPbIqV92AaeXatLxBI9gBaebbnrfifHhDYfgasaacH8akY=wiFfYdH8Gipec8Eeeu0xXdbba9frFj0=OqFfea0dXdd9vqai=hGuQ8kuc9pgc9s8qqaq=dirpe0xb9q8qiLsFr0=vr0=vr0dc8meaabaqaciaacaGaaeqabaqabeGadaaakeaadaaeWbqaaiabdEha3naaBaaaleaacqWGPbqAcqWGLbqzaeqaaaqaaiabdMgaPjabg2da9iabigdaXaqaaiabdYeambqdcqGHris5aOGaeyizImQaem4saSKaeyipaWZaaabCaeaacqWG3bWDdaWgaaWcbaGaemyAaKMaemyzaugabeaaaeaacqWGPbqAcqGH9aqpcqaIXaqmaeaacqWGmbatcqGHRaWkcqaIXaqma0GaeyyeIuoakiabc6caUiaaxMaacaWLjaWaaeWaaeaacqaIXaqmcqaIXaqmaiaawIcacaGLPaaaaaa@4DEF@

In the second step, we take the imputed value as a linear combination of the original value and the value suggested by the neighbours

x′re
 MathType@MTEF@5@5@+=feaafiart1ev1aaatCvAUfKttLearuWrP9MDH5MBPbIqV92AaeXatLxBI9gBaebbnrfifHhDYfgasaacH8akY=wiFfYdH8Gipec8Eeeu0xXdbba9frFj0=OqFfea0dXdd9vqai=hGuQ8kuc9pgc9s8qqaq=dirpe0xb9q8qiLsFr0=vr0=vr0dc8meaabaqaciaacaGaaeqabaqabeGadaaakeaacuWG4baEgaqbamaaBaaaleaacqWGYbGCcqWGLbqzaeqaaaaa@311D@ = *w*_re_*x*_re _+ (1 - *w*_re_)x^re
 MathType@MTEF@5@5@+=feaafiart1ev1aaatCvAUfKttLearuWrP9MDH5MBPbIqV92AaeXatLxBI9gBaebbnrfifHhDYfgasaacH8akY=wiFfYdH8Gipec8Eeeu0xXdbba9frFj0=OqFfea0dXdd9vqai=hGuQ8kuc9pgc9s8qqaq=dirpe0xb9q8qiLsFr0=vr0=vr0dc8meaabaqaciaacaGaaeqabaqabeGadaaakeaacuWG4baEgaqcamaaBaaaleaacqWGYbGCcqWGLbqzaeqaaaaa@3121@.     (12)

As for weighted reporter average above, when the quality weight is zero, we ignore the original value. When the weight is unity, we trust the original value and ignore the value suggested by the neighbours.

#### LSimpute

Bø *et al*. showed that LSimpute_adaptive is a very good method for imputation of missing values [17]. The method is based on the least squares principle, which means the sum of squared errors of a regression model is minimised and the regression model is used to impute missing values. The method utilises correlations both between reporters and experiments.

In the comparisons made in this report, we used the LSimpute_adaptive algorithm implemented in the publicly available LSimpute program [17].

### Evaluation method

In order to validate the imputation methods we did as follows for each of the three data sets. We split the data into replicate data sets: two sets for the melanoma and breast cancer data, and four sets for the mycorrhiza data. We imputed the data in one of the replicate data sets and compared the imputed data, *x*', to the other pristine replicate data, *y*. For the mycorrhiza data, we compared the imputed data to the (non-weighted) average of the three pristine replicate data sets. We measured the quality of the method using the mean squared deviation

MSD=1N∑i=1N(x′i−yi)2.     (13)
 MathType@MTEF@5@5@+=feaafiart1ev1aaatCvAUfKttLearuWrP9MDH5MBPbIqV92AaeXatLxBI9gBaebbnrfifHhDYfgasaacH8akY=wiFfYdH8Gipec8Eeeu0xXdbba9frFj0=OqFfea0dXdd9vqai=hGuQ8kuc9pgc9s8qqaq=dirpe0xb9q8qiLsFr0=vr0=vr0dc8meaabaqaciaacaGaaeqabaqabeGadaaakeaacqqGnbqtcqqGtbWucqqGebarcqGH9aqpdaWcaaqaaiabigdaXaqaaiabd6eaobaadaaeWbqaaiabcIcaOiqbdIha4zaafaWaaSbaaSqaaiabdMgaPbqabaGccqGHsislcqWG5bqEdaWgaaWcbaGaemyAaKgabeaakiabcMcaPmaaCaaaleqabaGaeGOmaidaaOGaeiOla4IaaCzcaiaaxMaadaqadaqaaiabigdaXiabiodaZaGaayjkaiaawMcaaaWcbaGaemyAaKMaeyypa0JaeGymaedabaGaemOta4eaniabggHiLdaaaa@496B@

where the sum runs over all expression values in all replicate data sets, except spots in the pristine data set that were marked as invalid in the data pre-processing step described above. The fraction of spots not used in the summation were: 6% for the melanoma data, 7% for the breast cancer data, and 8% for the mycorrhiza data.

The motivation for this choice of MSD as evaluation metric is threefold. First, in the weighted methods the imputed value is a linear combination of the value suggested by the neighbours and the original value. Hence, comparing with the original value would introduce an information leak, making the evaluation unfair. Second, introducing artificial missing values randomly may not be optimal [15,25], since it assumes missing values to occur uncorrelated. By using replicates we could avoid this problem and mark spots as missing values depending on their quality. Third, we avoided any bias that could be introduced by imputing both replicates and comparing the imputed values. By considering the zero impute method (missing values are set to zero), it is easy to understand that a bias could be introduced. If both replicate spots are imputed, *i.e*., both set to zero, they would have no deviation and the evaluation would obviously be flattering.

## Availability and requirements

Project name: WeNNI

Project home page: 

Operating system: All that supports GCC 4.0 or later (tested on GNU/Linux and MacOS X 10.4).

Other requirements: Gnu Scientific Library, GSL. 

License: GNU General Public License

## Authors' contributions

Both authors developed weighted methods, designed and performed comparisons of methods, and wrote the manuscript.

## Figures and Tables

**Table 1:** Comparisons of WeNNI, KNNimpute, and LSimpute adaptive using two different measures. MSD is the mean squared deviation calculated over all spots, whereas MSD_imputed is calculated over spots with SNR smaller than *β*, *i.e*., the spots imputed in non-weighted methods. *β *was chosen to yield the lowest MSD for LSimpute adaptive. WeNNI is more accurate than LSimpute and KNNimpute, even though *β *was tuned to optimise the performance of LSimpute.

Data set	Measure	*β*	WeNNI	KNNimpute	LSimpute adaptive
*Breast cancer*	MSD	0.2	0.345	0.369	0.368
	MSD_imputed		1.59	1.81	1.75
*Melanoma*	MSD	0.6	0.995	1.08	1.05
	MSD_imputed		3.41	3.77	3.64
*Mycorrhiza*	MSD	0.2	0.216	0.241	0.244
	MSD_imputed		0.840	0.902	0.954
